# The effect of Cu loading on the selective catalytic reduction of NO_*x*_ in diesel exhaust over SAPO-34

**DOI:** 10.1039/d6ra02407f

**Published:** 2026-09-02

**Authors:** He Huang, Zifei Ni, Shujun Yan, Heyuan Tian

**Affiliations:** a School of Transportation Engineering, Nanjing University of Industry Technology Nanjing 210023 China 2018100903@niit.edu.cn +86-025-85864356; b College of Chemical Engineering and Environment, State Key Laboratory of Heavy Oil Processing, China University of Petroleum-Beijing Beijing 102249 China

## Abstract

Ammonia selective catalytic reduction (NH_3_-SCR) is currently the most effective technical approach for NO_*x*_ removal from exhaust gases. In this study, Cu_*x*_-SAPO-34 catalysts were prepared by a two-step ion-exchange method using H-SAPO-34 molecular sieves as the support, and the effect of copper loading on the NH_3_-SCR performance of Cu_*x*_-SAPO-34 catalysts was systematically investigated. The NH_3_-SCR performance evaluation device was set up to explore the influence of different copper loadings on the catalyst performance. Multiple characterization techniques, including XRD, ICP, XPS, BET, H_2_-TPR, and NH_3_-TPD, were employed to study the impact of the physical structure and chemical properties of Cu_*x*_-SAPO-34 catalysts on NH_3_-SCR performance from a microscopic perspective. Additionally, first-principles calculation methods were used to systematically investigate the evolution laws of the SAPO-34 support structure, Cu species morphology, active site characteristics, and NH_3_-SCR reaction pathways under the regulation of Cu loading. The results show that Cu_4_-SAPO-34 demonstrates superior denitrification activity at lower temperature ranges compared to the Cu_2_-SAPO-34 and Cu_6_-SAPO-34 samples, and the conversion rate increases with the increase in temperature, reaching a maximum of 98.66% at 170 °C and gradually decreasing from 360 °C. The NH_4_-SAPO-34 has a larger specific surface area, which shows a trend of first increasing and then decreasing with the increase in Cu content. The Cu loading does not destroy the framework structure of the molecular sieve, and the XRD diffraction peaks of all catalyst samples maintain the original chabazite (CHA) structure of the H-SAPO-34 molecular sieve. The Cu_4_-SAPO-34 has the largest total number and highest strength of acidic sites, and excessive Cu loading will cover or neutralize some acidic sites, leading to a decrease in acidity. The adsorption of NH_3_ is the first step of the entire reaction, and the calculation reveals that the Cu^2+^ site is the active site of the reaction.

## Abstract 2

A series of Cu-loaded SAPO-34 zeolites were synthesized for selective catalytic reduction of NO with NH_3_, and the redox cycle of Cu^2+^/Cu^+^ active sites during the catalytic process was clarified.

## Graphical abstract

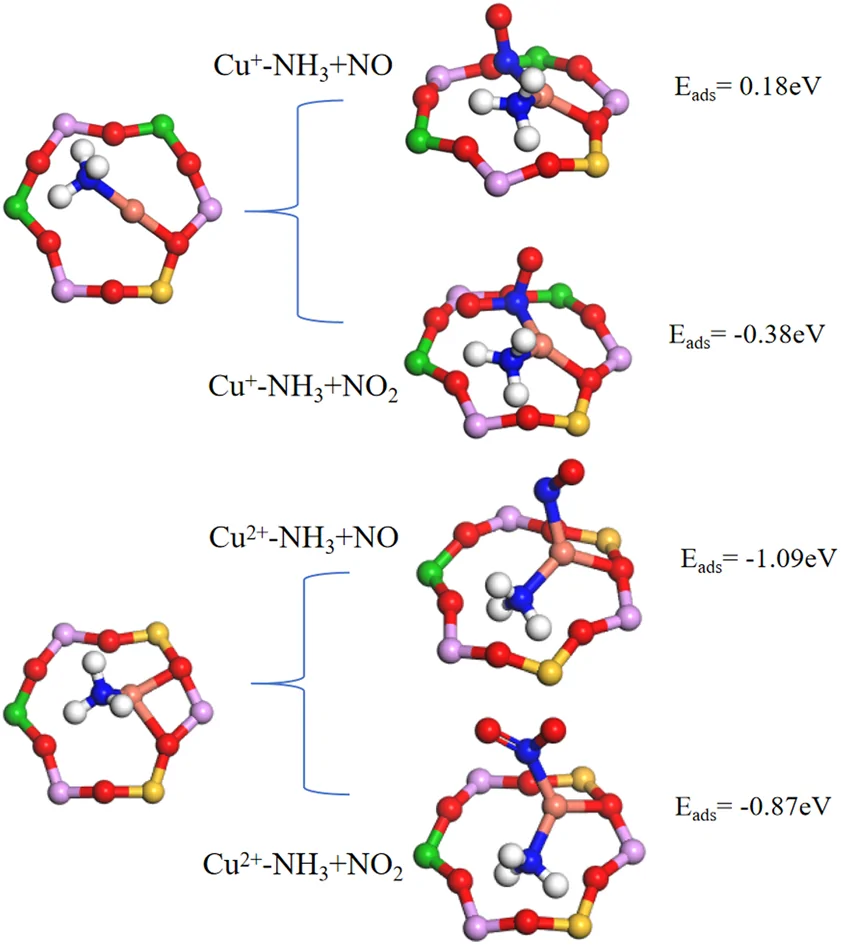

## Introduction

Diesel engines occupy a core position in the fields of diesel vehicles, engineering machinery and marine power due to their high thermal efficiency and strong reliability.^1^ However, nitrogen oxides (NO_*x*_) generated during combustion are major atmospheric pollutants. Their emissions give rise to environmental problems including photochemical smog and acid rain, while also posing threats to the human respiratory system. These issues have emerged as global challenges in environmental governance.^2,3^ To address pollution, China's “National VI” emission standards have set stringent limits on NO_*x*_ emission from exhaust gases, requiring heavy-duty diesel vehicles to reduce NO_*x*_ emissions to 0.02 g (kW h)^−1^, making it difficult for traditional exhaust purification technologies to meet regulatory demands.^4–6^

Ammonia selective catalytic reduction (NH_3_-SCR) is still the main technological pathway for NO_*x*_ removal from exhaust gases.^7^ The development of NH_3_-SCR catalysts has mainly gone through precious metals, metal oxides, and then the molecular sieve type.^8–10^ Precious metal catalysts were the first SCR catalysts used. And these show a low operating temperature but are very active.^11^ However, due to their high oxidation potential, they are prone to side reactions, and their high cost hinders large-scale industrial application. Therefore, these catalysts have gradually been replaced by new metal oxide and molecular sieve catalysts.^12,13^ Metal oxides, particularly transition metal oxides, have attracted widespread attention in SCR denitrification applications due to their variable valence characteristics, low cost, environmental friendliness, and easy-to-control properties.^14^ Currently, research on transition metal oxide catalysts mainly focuses on Ce-based, Mn-based, and Fe-based metal oxides, which are often used as the main active components of catalysts due to their variable valence states and excellent redox properties.^15–17^ He *et al.*^18^ used an impregnation method to load different amounts of CeO_2_ onto a TiO_2_ support, demonstrating excellent SCR performance and N_2_ selectivity. Liu *et al.*^19^ made several different kinds of Mn–Ti oxides through sol–gel, and they looked at how the various preparation conditions influenced catalyst performance. From this, we found that the calcination temperature and metal content greatly influenced the catalyst and SCR performance. Yao *et al.*^20^ made different kinds of MnO_*x*_-CeO_2_ catalysts and found out that the coprecipitated catalyst was the most efficient at more than 90% NO_*x*_ conversion at 100–325 °C with over 70% N_2_ selectivity at 100–200 °C. Shi *et al.*^21^ enhanced the catalyst's resistance to SO_2_ and H_2_O poisoning by modulating the microstructure of MnCoO_*x*_, while also strengthening the catalyst's resistance to alkali metal poisoning.

Molecular sieves, due to their abundant acidic sites, unique pore structures, high specific surface areas and excellent thermal stability, are also commonly used as catalyst supports and have great application potential in SCR catalysts.^22–24^ Currently, the molecular sieves mainly used as NH_3_-SCR catalyst supports include ZSM-5, beta and CHA, with the most studied active components being Cu and Fe. Among them, Cu-based molecular sieve catalysts have attracted significant attention because of their higher low-temperature SCR performance and easy formation by the Cu^2+^ ion, which can form a copper-quaternary ammonium salt complex ion to go through the molecular sieve pores leading to isolated Cu^2+^ ions. This has sparked a research boom.^25–27^ Among copper-based microporous molecular sieves, Cu-CHA, Cu-LTA and Cu-AEI are the most extensively studied structures. Torre *et al.*^28^ prepared Cu-beta molecular sieve catalysts and found that their denitrification activity temperature range is between 300–500 °C. Li *et al.*^29^ prepared Fe-MOR catalysts, achieving NO_*x*_ conversion rates of 80% at 300–500 °C. Chen *et al.*^30^ prepared Fe-MCM-22 catalysts, achieving over 80% NO_*x*_ conversion at 200–500 °C. Cu-beta and Fe-beta molecular sieve catalysts have become new research hotspots due to their higher hydrothermal stability, following ZSM-5 type molecular sieve catalysts.^31,32^ CHA-type molecular sieves have become a research focus as NH_3_-SCR catalyst supports in recent years. Sun *et al.*^33^ proposed a reconstruction synthesis strategy using Cu-amine complexes as precursors and Cu sources to prepare Cu-SAPO-34 catalysts through reconstruction reactions. Also has the advantages of having a wide crystallization phase range and a large amount of solid yield and a controllable Si/Cu ratio as well as homogenous distribution of the Cu species over the molecular sieve cavities. After high-temperature hydrothermal treatment, it can still maintain a high content of isolated Cu^2+^, demonstrating excellent catalytic activity and hydrothermal stability.

Density Functional Theory (DFT), as a core method in quantum chemical calculations, has become an important auxiliary tool in the study of Cu-based molecular sieve NH_3_-SCR catalysts due to its ability to reveal the microscopic mechanism of catalytic reactions from atomic and electronic levels and accurately predict the structure–performance relationships of catalysts.^34–36^ DFT calculations can simulate the adsorption configurations of various intermediates, reaction energy barriers, and transition state structures during the reaction process, accurately analyzing the dominant reaction mechanisms and rate-controlling steps under different conditions and catalyst systems, compensating for the limitations of experimental methods in capturing reaction intermediates and transition states, and providing important theoretical support for the refinement of reaction mechanisms. For the Cu/SAPO-34 catalyst, DFT calculations clarified the evolution of the NH_3_-SCR reaction mechanism at different Cu loadings. At low Cu loading, the reaction mainly follows the Eley–Rideal mechanism, where NH_3_ molecules adsorbed on Brønsted acid sites (forming NH_4_^+^) react with gas-phase NO, with the rate-controlling step being the dehydrogenation of NH_4_^+^, and the reaction energy barrier is 1.2–1.5 eV. Isolated Cu^2+^ can lower this barrier to 0.8–1.0 eV, thereby enhancing low-temperature activity.^37–39^ At suitable Cu loading, the reaction exhibits characteristics of a synergistic effect between the Langmuir–Hinshelwood and Eley–Rideal mechanisms, where isolated Cu^2+^ sites activate NO, and Brønsted acid sites adsorb NH_3_, leading to the formation of the intermediate NH_4_NO_2_ at the active site interface, which subsequently decomposes into N_2_ and H_2_O. At this point, the redox cycle barrier of Cu^2+^/Cu^2+^ is only 0.6–0.9 eV, and the overall reaction barrier is reduced to 0.7–1.1 eV, resulting in optimal catalytic activity.^40,41^ At high Cu loading, the dominant reaction mechanism shifts to the Langmuir–Hinshelwood mechanism, where CuO clusters adsorb NO and NH_3_ to generate the unstable intermediate NH_4_NO_3_, with a decomposition barrier as high as 1.8–2.2 eV, while CuO clusters promote the non-selective oxidation of NH_3_, thereby reducing the selectivity of N_2_.^42–44^

This study uses H-SAPO-34 molecular sieves as supports and employs a two-step ion exchange method to prepare Cu_*x*_-SAPO-34 catalysts. The effect of Cu loading on the NH_3_-SCR performance of Cu_*x*_-SAPO-34 catalysts was systematically studied. The physicochemical properties were investigated by X-ray photoelectron spectroscopy (XPS), X-ray diffraction (XRD), Brunauer–Emmett–Teller (BET) adsorption isotherms, and H_2_ temperature-programmed reduction (H_2_-TPR). Under simulated diesel exhaust conditions, the NH_3_-SCR performance was evaluated using a fixed-bed reactor. This article systematically investigates the evolution of the structure of the SAPO-34 support, the morphology of Cu species, the characteristics of active sites, and the reaction pathways of NH_3_-SCR under the regulation of Cu loading using MS software and experimental characterization methods. This work offers significant theoretical support for the rational design and performance optimization of catalysts. The findings of the present work provide meaningful insights into the structure–activity relationship of Cu_*x*_-SAPO-34 catalysts, and serve as a useful reference for the rational design of high-efficiency SCR catalysts for NO_*x*_ abatement from diesel engines.

## Experimental

### Catalyst preparation

Cu/SAPO-34 catalysts with the Cu content of 2 wt%, 4 wt% and 6 wt% were prepared utilizing a two-step ion exchange method.

In the first step, the H-SAPO-34 molecular sieve underwent ammonium exchange. First, 5 g of H-SAPO-34 molecular sieve was weighed and placed in a beaker with 50 mL of ammonium nitrate solution, followed by the addition of 200 mL of deionized water and allowed to stand for a period of time. Subsequently, it was placed in a magnetic stirring water bath at 80 °C for 2 hours of constant temperature stirring. Subsequently, the mixture was filtered and washed thoroughly with deionized water. The resulting filter cake was dried in an oven at 100 °C for 10 h to afford the NH_4_-SAPO-34 molecular sieve.

NH_4_-SAPO-34 powder and 0.3 g, 0.6 g and 0.9 g copper nitrate crystals were placed in a beaker, 200 mL of deionized water was added, and then left to stand for a period of time. It was then placed in a magnetic stirring water bath, stirred vigorously at 80 °C constant temperature water bath for 2 hours, and then filtered and washed sufficiently with deionized water. After drying in an oven at 100 °C for 10 hours, it was calcined in a muffle furnace at 550 °C for 4 hours to obtain the Cu_*x*_-SAPO-34 catalyst. Depending on the copper loading, it was named Cu_*x*_-SAPO-34, where “*x*” stands for the certain mass percentage of copper in the end catalyst.

### Catalyst characterization

XRD characterization was carried out using a Japan Rigaku Miniflex X-ray diffractometer with a Cu Kα radiation source, operating at a tube voltage of 30 kV and a current of 15 mA, with a 2*θ* angle range of 3° to 60° and a step size of 0.02° for continuous scanning.

The content of each element in the catalyst wasanalyzed on an Agilent 5800 Inductively Coupled Plasma Optical Emission Spectrometer (ICP-OES), where the solid powder sample of the molecular sieve was subjected to acid digestion to convert it into a liquid.

XPS testing was conducted using a Thermo Scientific K-Alpha analyzer. The Al Kα beam (*hν* = 1486.8 eV) was used, which was adjusted with the C 1s peak (284.8 eV), the source voltage is set at 15 kV and current is fixed as 10 mAp, the scan resolution is taken to be 0.05 eV.

BET (Brunauer–Emmett–Teller) testing was conducted using a Micromeritics ASAP 2020 Automatic Physical Adsorption instrument from the US to determine specific surface area and pore size distribution. The surface area was calculated based on the BJH (Barrett–Joyner–Halenda) method and then the pore volume distribution curve is obtained by using BJH methodology. Before being tested it had been thermally processed at 300 °C for 5 hours.

H_2_-TPR was tested on the AutoChem II chemisorption apparatus. The exact process is as follows: 30 mg of the catalyst was placed in a u-shaped reactor; firstly it got heated up using Ar gas till 300 °C then cooled down to 50 °C. And then we injected a 10% H_2_/Ar gas. Then the material got heated up from 50 °C to 800 °C at a ramp of 10 °C min^−1^.

NH_3_-TPD analysis was performed in a fixed-bed reactor, using a portable infrared online detector model DX4000 to measure the release amount of NH_3_, with a catalyst dosage of 100 mg, and a gas flow of 300 mL min^−1^. The testing steps are as follows: firstly, the catalyst is filled into a 6 mm quartz reaction tube and pretreated at 360 °C under a flow of 300 mL min^−1^ of N_2_ for 1 h. Then, the temperature is reduced to 50 °C and a mixed gas containing 500 ppm NH_3_ at a flow rate of 300 mL min^−1^ is introduced into the catalyst for adsorption for 1 h. After that, the sample is purged with N_2_ for 1 h. Finally, the temperature is raised to 600 °C at a heating rate of 10 °C min^−1^ (Fig. 1).

**Fig. 1:**
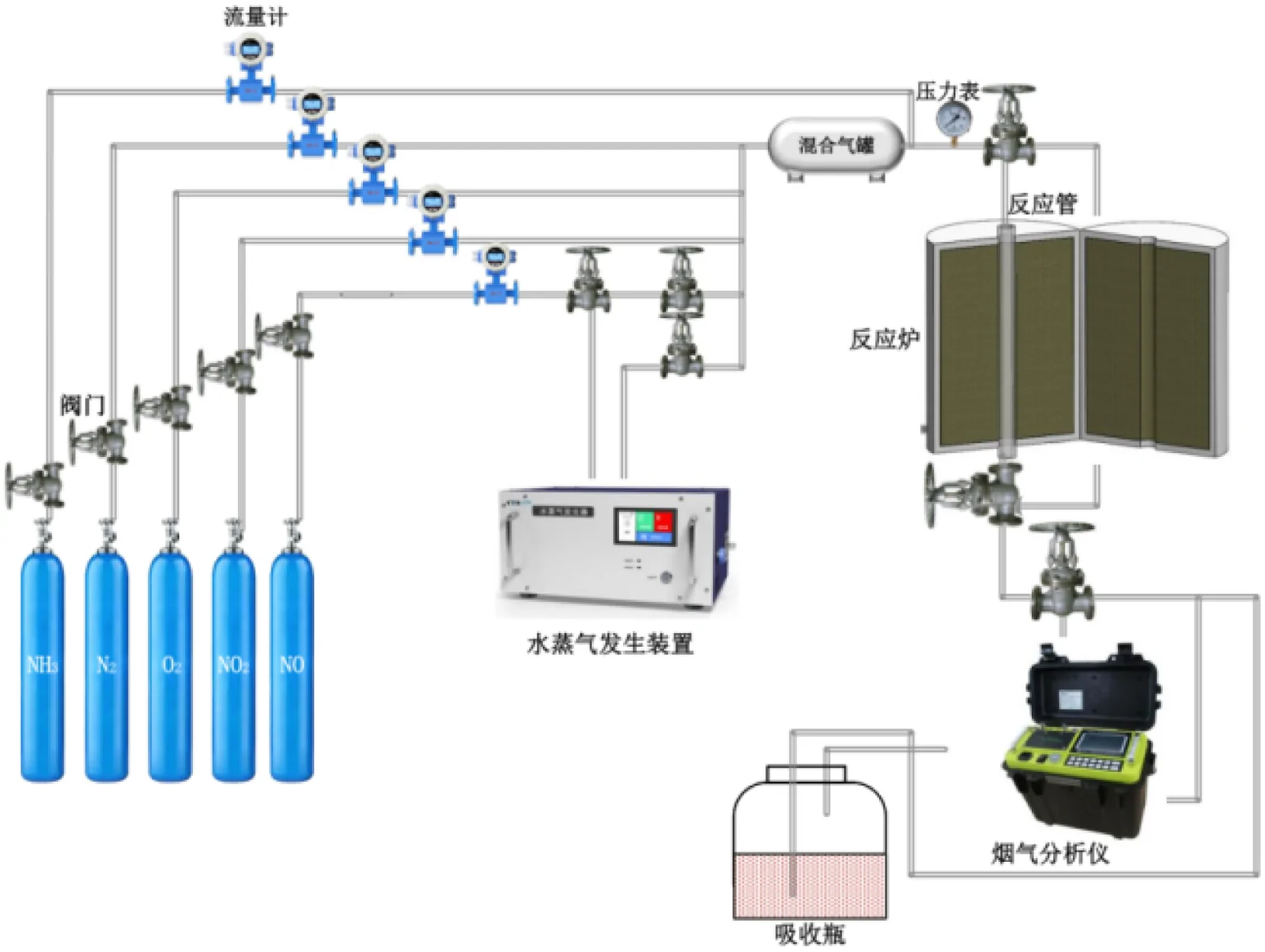
Diagram of SCR performance evaluation device.

### Evaluation of the NH_3_-SCR performance

Catalytic evaluations were performed in a 7 mm inner-diameter fixed-bed reactor. It particulate sample was loaded 40–60 mesh quartz tube for NH_3_-SCR performance testing. The gas fed into the reactor consisted of 500 ppm NO, 500 ppm NH_3_, 50 ppm N0_2_, 5% O_2_, and 5% H_2_O, with N_2_ used as the carrier gas. The volume flow rate was as high as 400 mL min^−1^, and the gas hourly space velocity (GHSV) was around 150 000 mL (g h)^−1^. A GasMet DX-4000 online gas analyzer was employed to monitor the exhaust gas composition, and the catalytic performance of the catalysts was determined at temperatures ranging from 150 to 500 °C. The NO_*x*_ conversion rate is calculated by the following formula:^45^1
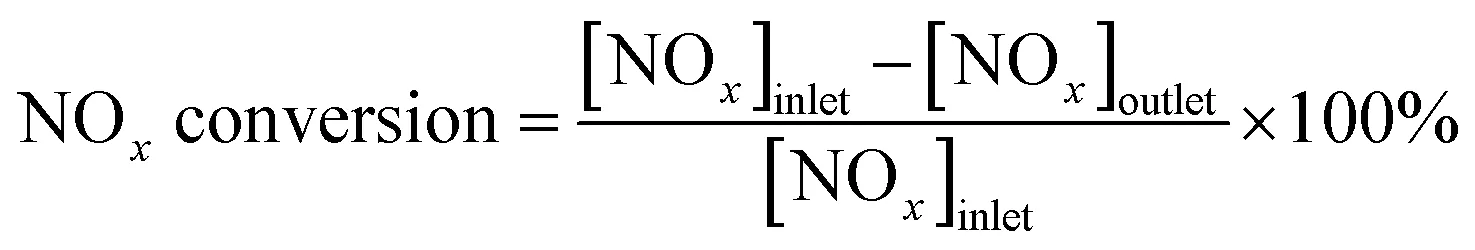


## Results and discussion

### Evaluation results of NH_3_-SCR catalytic activity

Fig. 2 shows the de-NO_*x*_ activity of three catalysts in the temperature range of 120 to 490 °C. As can be seen from the figure, the conversion rates of the three catalysts exhibit a “volcanic” pattern, meaning that the catalytic activity increases with the increase of Cu loading, but when the Cu loading reaches a certain level, the catalytic activity begins to decrease in a regular manner. At the same time, it can be seen that the Cu loading has a greater impact on the low-temperature reaction stage by comparing the changes in the NO_*x*_ conversion rates of the three catalysts during the low-temperature and high-temperature reaction stages. It can also be observed that the Cu loading has a greater impact on the low-temperature reaction stage. The figure also shows that the de-NO_*x*_ activity of the Cu_2_-SAPO-34 catalyst is the lowest across the entire test temperature range. The NO_*x*_ conversion rate gradually increases with the rise in temperature, starting from 120 °C, reaching the highest at 390 °C, which is 95.65%. In contrast, the Cu_4_-SAPO-34 catalyst exhibits good low-temperature activity for NO_*x*_, with a conversion rate exceeding 70% at 130 °C, and the conversion rate also increases with temperature, reaching a maximum of 98.66%, before gradually decreasing after 360 °C.

**Fig. 2:**
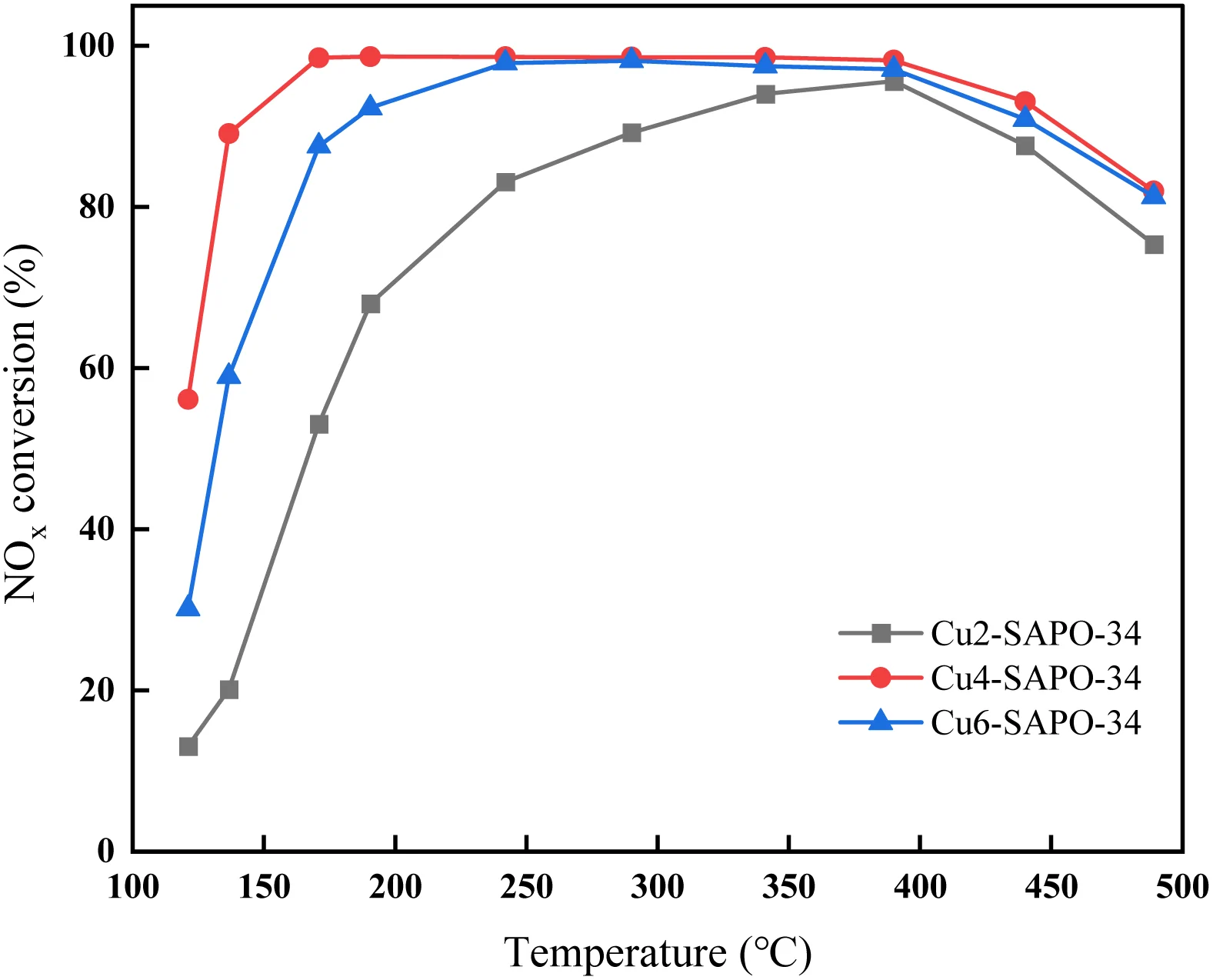
The nitrogen oxide conversion rate of Cu_*x*_-SAPO-34 catalyst.

### Structural analysis

#### Texture structure (BET and ICP)

Table 1 shows the pore volume and specific surface area results of NH_4_-SAPO-34 and Cu_*x*_-SAPO-34 sample. As can be seen from Table 1, the pore diameters of the prepared samples are all less than 2 nm, exhibiting microporous characteristics. The abundance of micropores facilitates the transport of reactants and products, allowing the catalyst to demonstrate excellent de-NO_*x*_ activity. In addition, NH_4_-SAPO-34 has a relatively large specific surface area, which is beneficial for the uniform loading of the active component Cu ions on the molecular sieve, thereby promoting its catalytic performance more effectively. The specific surface area shows a trend of first increasing and then decreasing with the increase of Cu content, which is due to the excessive Cu accumulating on the support and blocking the channels of the molecular sieve.

**Table 1:** The specific surface area and pore structure properties of Cu_*x*_-SAPO-34 catalyst

Catalyst	Surface area (m^2^ g^−1^)	Total pore volume (cm^3^ g^−1^)	Average pore diameter (nm)
NH_4_-SAPO-34	771.97	0.28	1.61
Cu_2_-SAPO-34	687.94	0.25	1.61
Cu_4_-SAPO-34	694.28	0.26	1.57
Cu_6_-SAPO-34	628.25	0.20	1.65

From the Cu/Al ratios of the surface and bulk phases of each catalyst in Table 2, it can be observed that as the copper loading increases, Cu species begin to accumulate more noticeably on the catalyst surface, indicating that surface copper oxide gradually becomes the main copper species in the Cu_*x*_-SAPO-34 catalyst.

**Table 2:** Surface and bulk composition analysis of Cu_*x*_-SAPO-34 catalyst

Catalyst	Surface Cu/Al mass ratio^a^ (%)	Body phase Cu/Al mass ratio^b^ (%)
Cu_2_-SAPO-34	1.3	2.32
Cu_4_-SAPO-34	1.9	2.95
Cu_6_-SAPO-34	2.1	3.14

a Calculated from the XPS results.

b Calculated from the ICP-OES results.

#### XRD analysis

Fig. 3 provides the XRD diagrams for H-SAPO-34 zeolite and different kinds of catalysts. As shown in the figure, after ion exchange, the XRD diffraction peaks of all catalyst samples maintain the original structure of H-SAPO-34 molecular sieve, which is the rhombohedral zeolite (CHA) structure. Compared to the H-SAPO-34 molecular sieve, it can be clearly seen that as the Cu content increases, the peak intensity of the catalyst gradually decreases, possibly due to excessive Cu species affecting the crystallization process of the molecular sieve. In addition, after the introduction of copper, the positions of the diffraction peak shift slightly to smaller angles, indicating that some copper atoms have entered the channels and lattice of SAPO-34 after ion exchange, leading to the expansion of the SAPO-34 molecular sieve lattice.

**Fig. 3:**
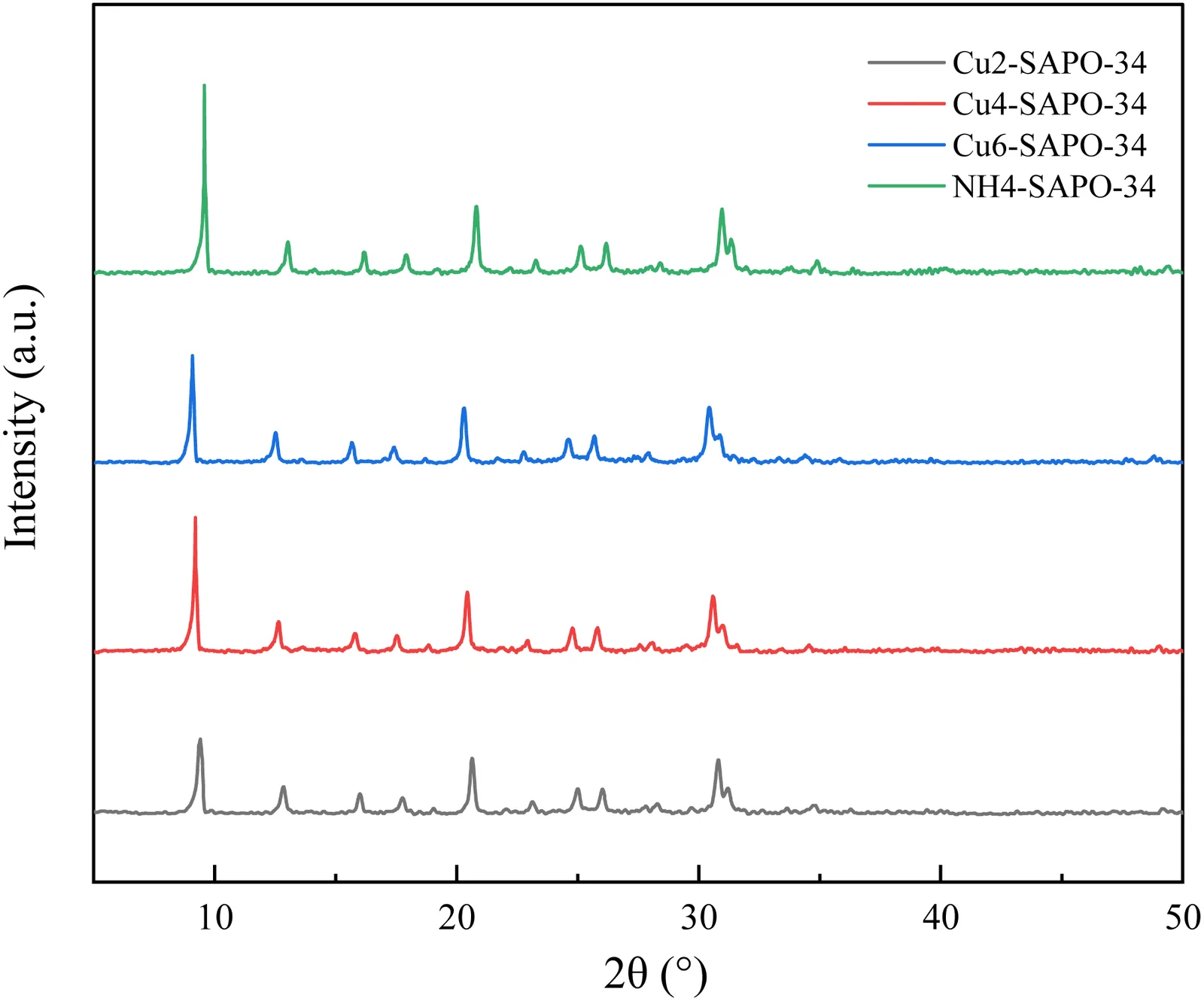
The XRD patterns of Cu_*x*_-SAPO-34 catalyst.

### Redox properties

#### XPS analysis

Fig. 4 shows the XPS survey spectrum of the Cu_*x*_-SAPO-34 catalyst. The characteristic peaks of SAPO-34 molecular sieve framework elements, including Al 2p, Si 2p, P 2p, and O 1s, are clearly observed for all three samples, indicating that the framework structure of the molecular sieve carrier remains intact after different Cu doping modifications, consistent with the XRD results. The presence of Cu 2p characteristic peaks in the high-binding-energy region confirms the successful introduction of copper species onto the surface of the molecular sieve material.

**Fig. 4:**
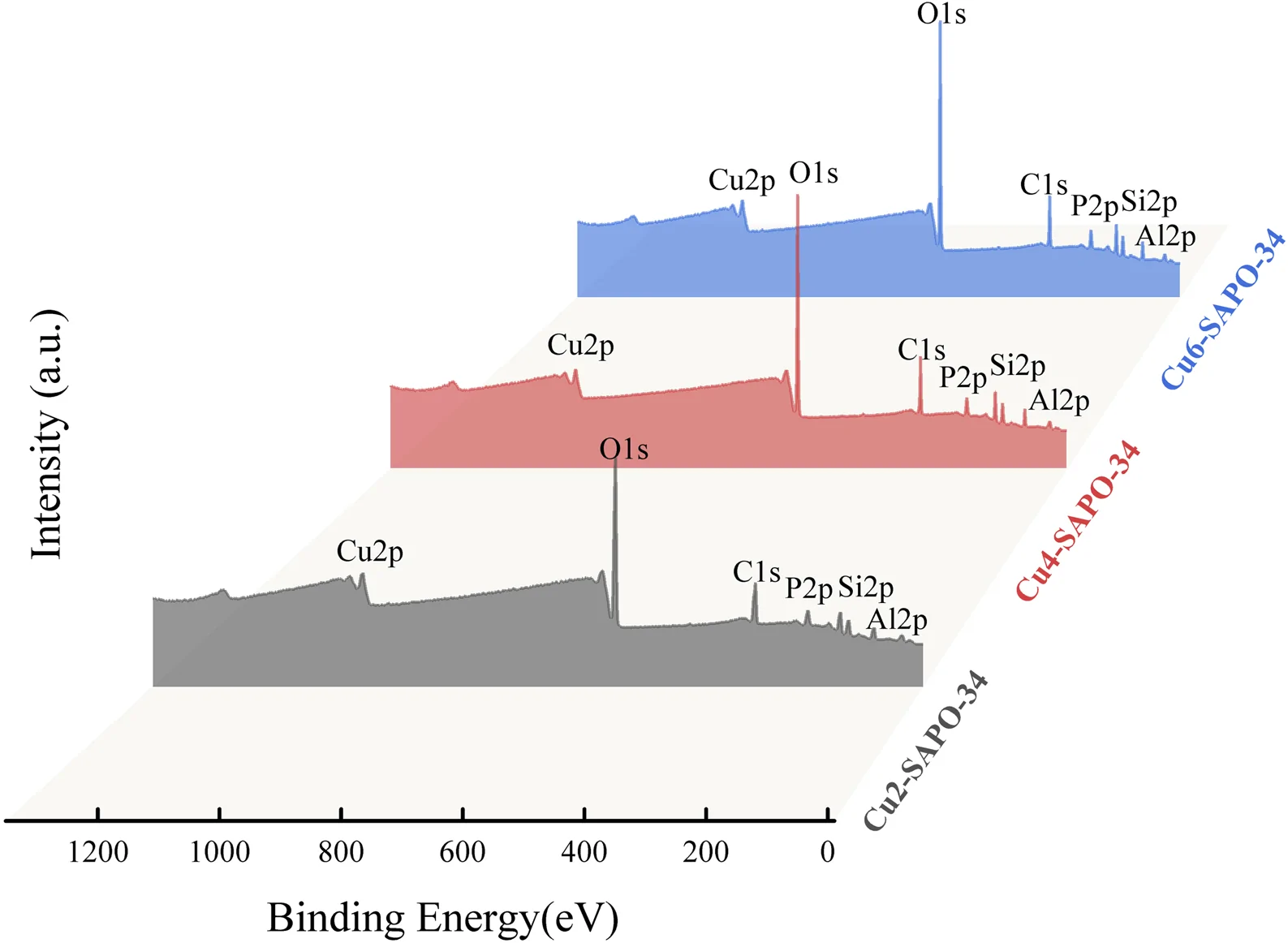
XPS survey spectrum of the Cu_*x*_-SAPO-34 catalyst.

Fig. 5 shows the Cu 2p XPS spectrum of the Cu_*x*_-SAPO-34 catalyst. All samples exhibit characteristic Cu 2p_3/2_ and Cu 2p_1/2_ double peaks near 933 eV and 953 eV, corresponding to divalent copper species. As the Cu doping ratio increases, the intensity of the Cu 2p peaks progressively enhances, indicating a gradual increase in the relative surface concentration of Cu^2+^. The Cu_2_-SAPO-34 sample exhibits the weakest Cu 2p peak intensity, suggesting limited exposed active sites on its surface, resulting in the lowest NH_3_-SCR catalytic performance. In contrast, the Cu_4_-SAPO-34 sample demonstrates optimal dispersion of copper species on the support surface, with sufficient active sites and no significant CuO aggregation blocking pores, leading to the best denitrification activity. Although the Cu 2p signal of the Cu_6_-SAPO-34 sample is slightly higher than that of Cu_4_-SAPO-34, excessive copper tends to accumulate on the zeolite surface, forming bulk CuO clusters. This not only hinders reactant mass transfer but also promotes non-selective oxidation of ammonia as a side reaction, thereby reducing its overall catalytic performance compared to Cu_4_-SAPO-34, which is consistent with the NH_3_-SCR activity test results.

**Fig. 5:**
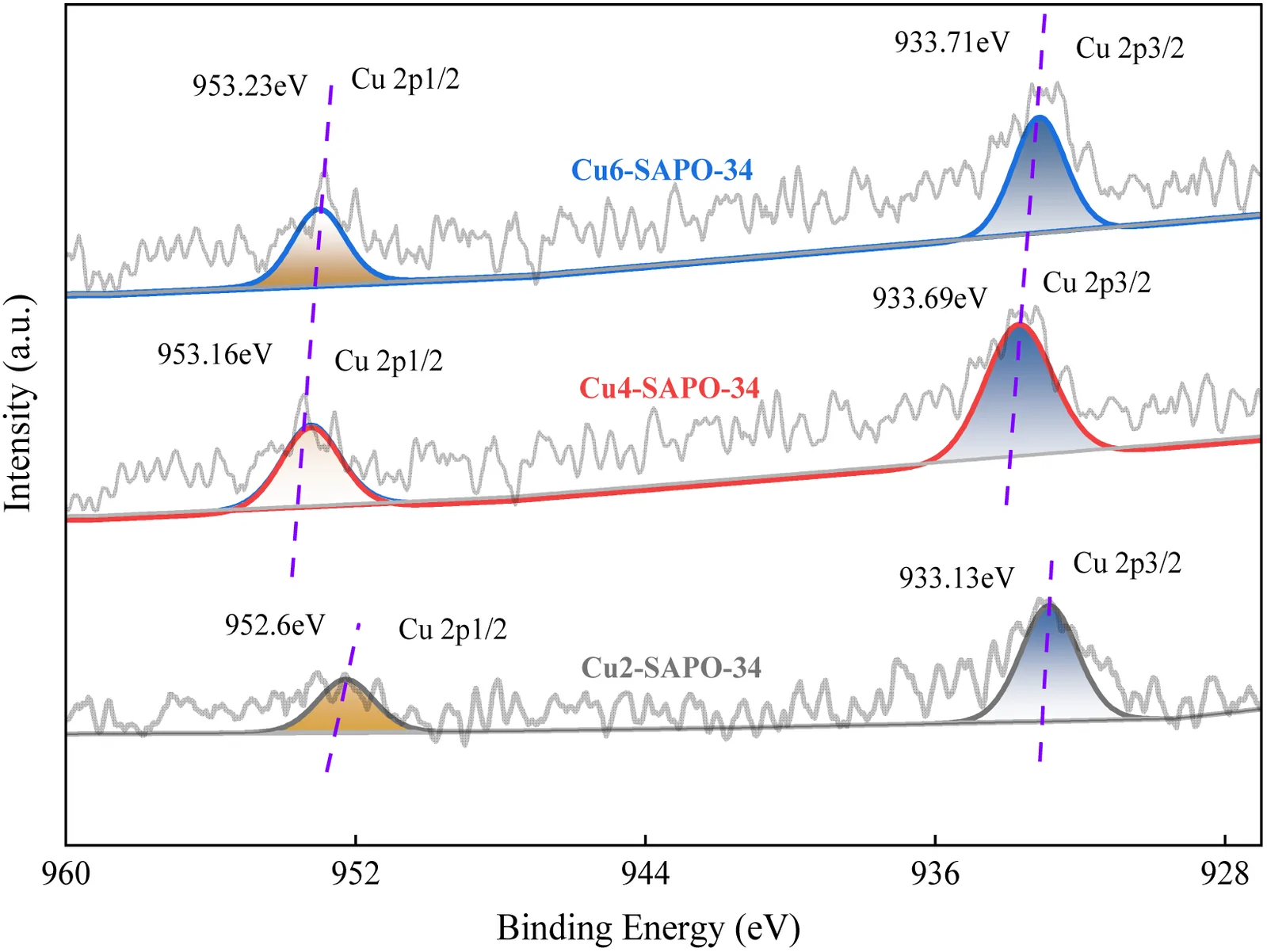
Cu 2p XPS spectrum of the Cu_*x*_-SAPO-34 catalyst.

The XPS analysis of the catalyst is given in Table 3. Comparing the Cu_*x*_-SAPO-34 catalyst with NH_4_-CHA, the contents of Al, P and Si are very similar, indicating that the skeletal element compositions of both are alike and belong to the CHA topological structure of molecular sieves. This also suggests that the Cu loading and calcination process did not damage the skeletal structure of the molecular sieve. From Cu_2_-SAPO-34 to Cu_4_-SAPO-34, the surface Cu content significantly increases, indicating that as the theoretical loading increases, the actual Cu deposited on the surface also increases. However, from Cu_4_-SAPO-34 to Cu_6_-SAPO-34, the Cu content slightly decreases, which may be due to Cu agglomeration at high loadings, with some Cu entering the pore channels, as seen in the specific surface area.

**Table 3:** Elemental analysis of the catalyst

	Cu_2_-SAPO-34	Cu_4_-SAPO-34	Cu_6_-SAPO-34	NH_4_-CHA
Al 2p	13.78	14.33	13.94	13.63
Si 2p	1.92	2.16	2.00	2.21
P 2p	11.17	11.09	11.34	11.13
Cu 2p	0.18	0.30	0.27	0

#### H_2_-TPR analysis

Fig. 6 shows the H_2_-TPR curves of the Cu_*x*_-SAPO-34 samples. From this diagram, it can be seen from the figure that the reduction peak of the Cu_*x*_-SAPO-34 catalyst is higher than that of NH_4_-SAPO-34, indicating that during the temperature-programmed reduction process, some of the molecular sieve framework may have undergone hydrogenolysis. Comparing the reduction peak areas of the three catalysts, it can be clearly seen that as the Cu content increases, the reduction peak area first increases and then decreases. It's due to the fact that the peak value is mainly contributed by Cu in the catalyst; when the Cu content is increased, so too is the catalyst's performance. However, when the Cu loading reaches a certain value, the catalytic activity shows a decreasing trend, as seen in the specific surface area.

**Fig. 6:**
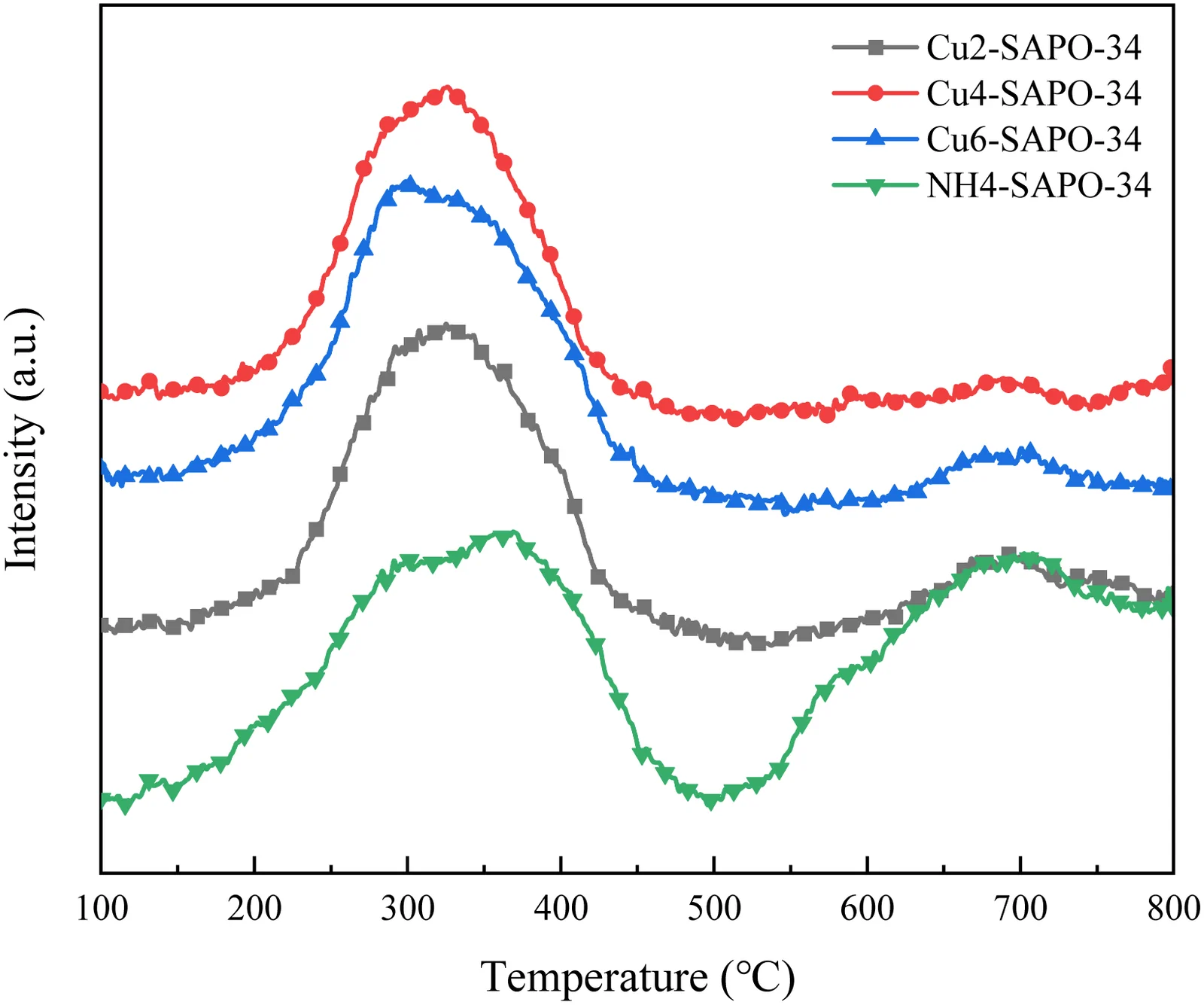
The H_2_-TPR curve of Cu_*x*_-SAPO-34 catalyst.

#### Acidity analysis (NH_3_-TPD)

Copper ion exchange effectively modulates the acidity of NH_4_-SAPO-34, significantly increasing both total acid amount and acid strength, as shown in Fig. 7. The total acidity of the copper-modified material is significantly higher than that of the parent NH_4_-SAPO-34. All catalysts in the figure exhibit two NH_3_ desorption peaks, where the low-temperature desorption peak at 100–200 °C corresponds to weak acidic sites, and the high-temperature desorption peak at 300–400 °C corresponds to medium-strong acidic sites. As the Cu loading increases, the center position of the low-temperature peak shifts slightly to the left, indicating that an increase in Cu loading slightly weakens the acidity of some weak acidic sites. Meanwhile, the center position of the high-temperature peak also shows a leftward shift, indicating that the number and strength of medium-strong acidic sites decrease with increasing Cu loading. Among the three catalysts prepared in this experiment, Cu_4_-SAPO-34 exhibits the highest total number of acidic sites and the best overall acid strength, whereas Cu_6_-SAPO-34 shows the lowest total acidity and acid strength. This suggests that excessive Cu loading in this system can cover or neutralize some acidic sites, leading to a decrease in acidity. Excessive Cu doping (Cu_6_-SAPO-34) leads to decreased total acidity, attributed to pore blockage and acid site coverage by aggregated CuO species.

**Fig. 7:**
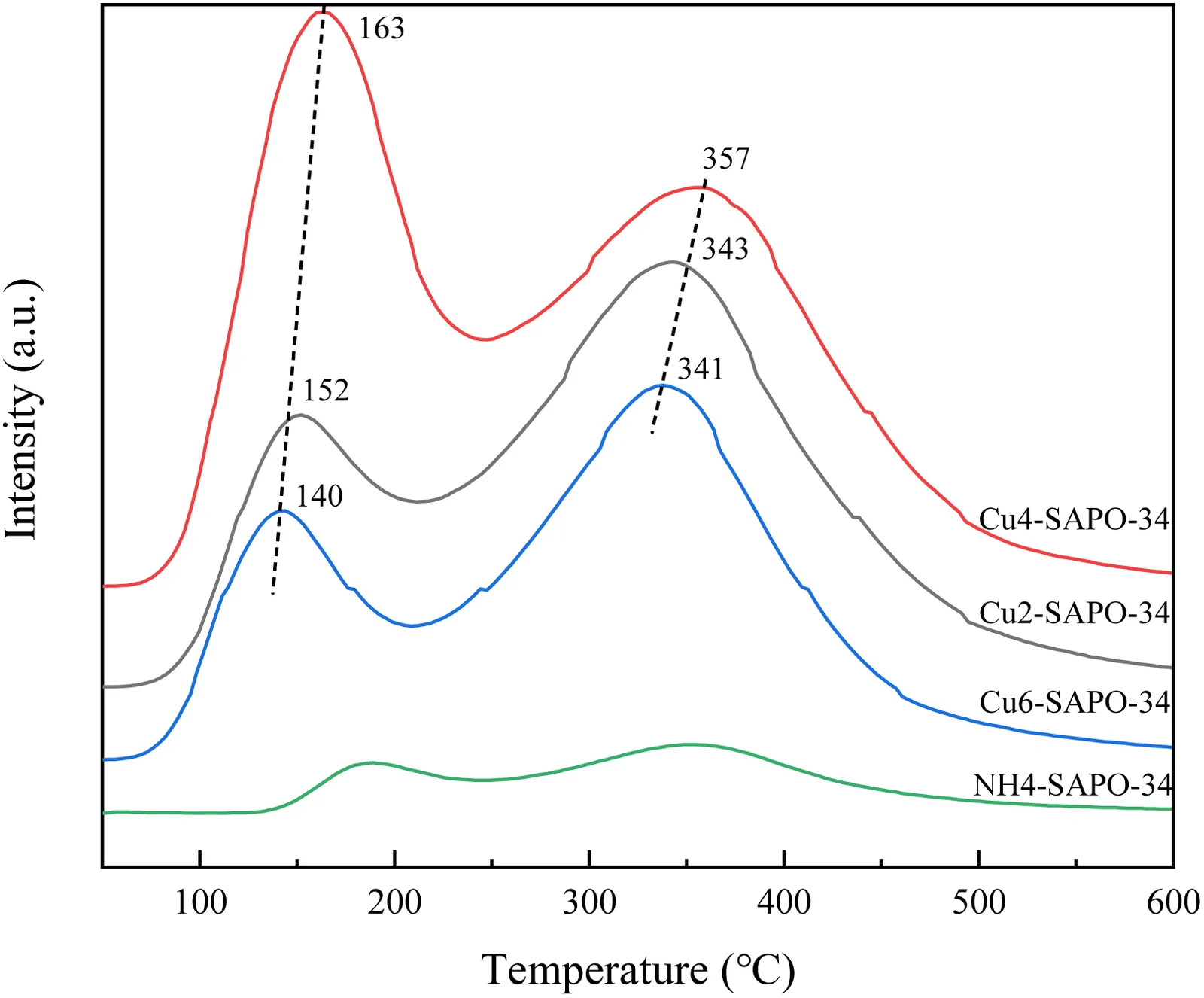
NH_3_-TPD profiles for Cu_*x*_-SAPO-34.

#### Reaction mechanism (numerical simulation)

In this study, all computational simulations were conducted using the CASTEP module of Materials Studio. The exchange–correlation energy was described by the GGA-PBE functional, and the projector augmented wave (PAW) method was used to represent the interaction between atomic nuclei and valence electrons. In preliminary lattice parameters setting, the plane wave cutoff energy was set at 400 eV. Structural relaxation continued until the Hellmann–Feynman force on all atoms were below 0.05 eV Å^−1^ and the energies of the self-consistent cycle were less than 10^−6^ eV. The parameter settings were verified before use to ensure the accuracy of the computational simulation results.

In the NH_3_-SCR reaction tests, due to the simultaneous presence of four gases, namely NO, NH_3_, NO_2_, and O_2_, their adsorption on copper ions was calculated to reveal the SCR reaction mechanism on Cu_*x*_-SAPO-34. By calculating the adsorption energy, it is possible to determine which gas molecules preferentially adsorb, thereby analyzing the reaction mechanism. The adsorption energy is represented as *E*_ads_, generally defined as: *E*_ads_ = *E*_slab+mol_ − (*E*_slab_ + *E*_mol_). Here, *E*_slab+mol_ stands for the total energy of the system after adsorption, *E*_slab_ represents the energy of the clean surface, and *E*_mol_ refers to the energy of the isolated gaseous molecule.

The structures of NH_3_, NO, NO_2_, and O_2_ adsorbed on Cu^+^ and Cu^2+^ are presented in Fig. 8, respectively, as (a) Cu^+^-NH_3_, (b) Cu^+^-NO, (c) Cu^+^-NO_2_, (d) Cu^2+^-NH_3_, (e) Cu^2+^-NO, and (f) Cu^2+^-NO_2_. The model of the studied molecule was established referring to the construction strategy described by Shi *et al.*^46^ From the results of the adsorption energy, it can be seen that, whether on Cu^+^ or Cu^2+^, the adsorption energy of NH_3_ molecules is the highest, and it is a chemical adsorption, indicating that the adsorption of NH_3_ is the first step of the entire reaction.

**Fig. 8:**
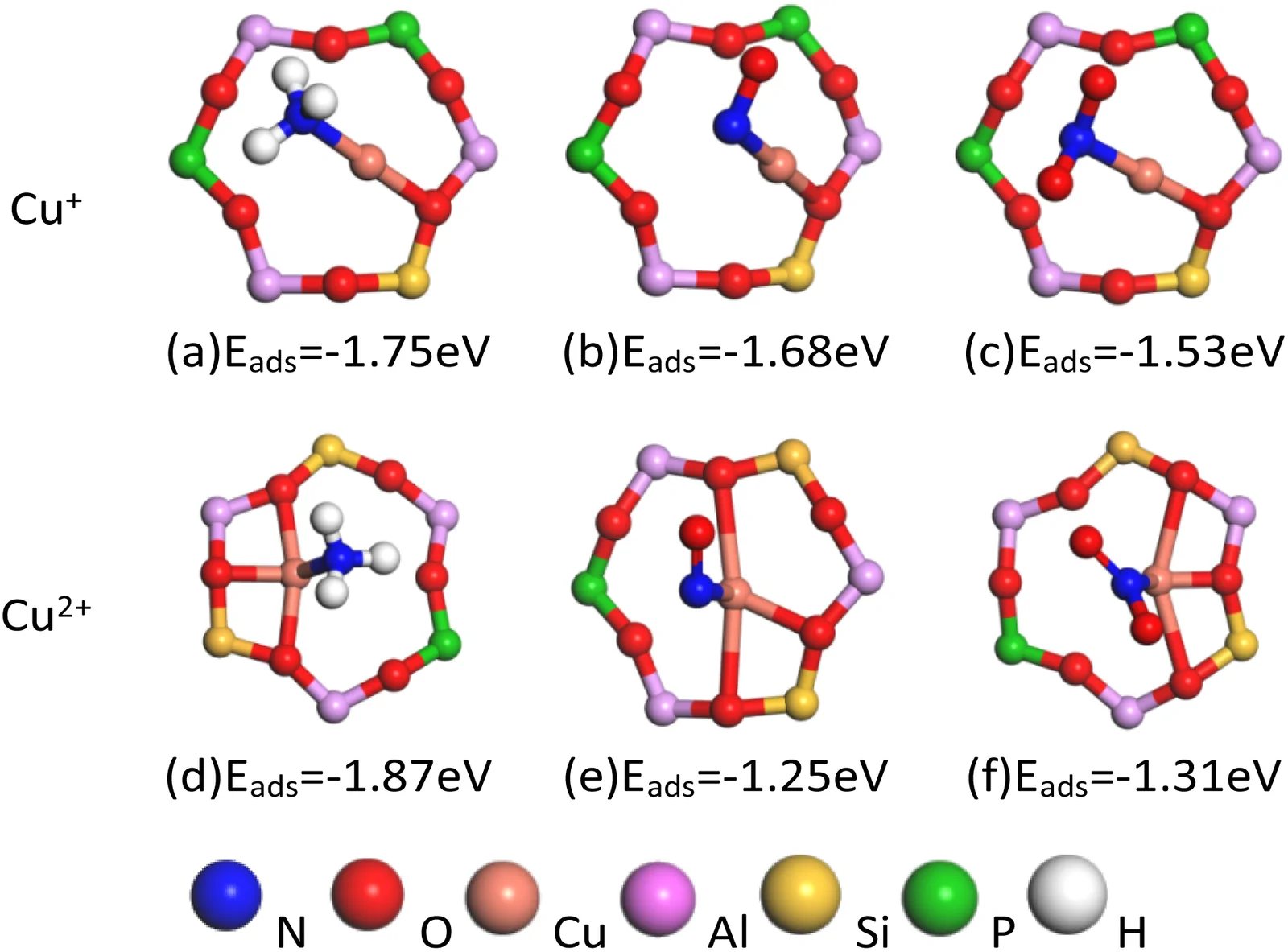
Structures of NH_3_, NO and NO_2_ adsorbed on Cu^+^ and Cu^2+^, show as (a) Cu^+^-NH_3_, (b) Cu^+^-NO, (c) Cu^+^-NO_2_, (d) Cu^2+^-NH_3_, (e) Cu^2+^-NO, (f) Cu^2+^-NO_2_.

The N atom in NH_3_ needs to combine with the N atom in NO or NO_2_ to generate N_2_. Therefore, the situation where NO and NO_2_ molecules are respectively co-adsorbed with NH_3_ on Cu^+^ and Cu^2+^ sites based on Cu^+^-NH_3_ and Cu^2+^-NH_3_ was further discussed. The adsorption configurations are illustrated in Fig. 9. Calculations revealed that the adsorption energy of NO on the Cu^+^-NH_3_ configuration is 0.18 eV, which is greater than zero, indicating a strong repulsive force between the NO molecule and the Cu^+^-NH_3_ surface. This means that NO cannot adsorb on Cu^+^-NH_3_, indicating that NH_3_ and NO cannot co-adsorb at the Cu^+^ site, which significantly hinders the generation of N_2_. The adsorption energy of NO_2_ on the Cu^+^-NH_3_ configuration is −0.38 eV, indicating that NH_3_ and NO_2_ can co-adsorb at the Cu^+^ site. On the Cu^2+^-NH_3_ configuration, the adsorption energies of NO and NO_2_ are −1.09 eV and −0.87 eV, respectively, indicating that NH_3_ and NO, as well as NH_3_ and NO_2_, can co-adsorb at the Cu^2+^ site, providing favorable conditions for the generation of N_2_. Therefore, in the SCR reaction, the Cu^2+^ site may be the active site of the reaction. NH_3_ molecules are adsorbed and activated at the Cu^2+^ active sites on the catalyst surface. Therefore, it can be inferred that the reaction process is as illustrated in Fig. 10, where NH_3_molecules are adsorbed and activated at the Cu^2+^ active sites on the catalyst surface. Different Cu loadings modulate the ratio of Cu^+^/Cu^2+^/CuO species, which not only affects NO_*x*_ conversion efficiency but also determines the strength of side reaction pathways; Cu^2+^ sites favor rapid N_2_ formation in standard SCR, while CuO aggregates serve as key sites for N_2_O byproduct generation. The activated NH_3_ reacts with NO in the gas phase to generate the key NH_2_NO intermediate. The intermediate decomposes into the final products N_2_ and H_2_O, while Cu^2+^ is reduced to Cu^+^. O_2_ in the gas phase re-oxidizes Cu^+^ back to Cu^2+^, completing the entire catalytic cycle.

**Fig. 9:**
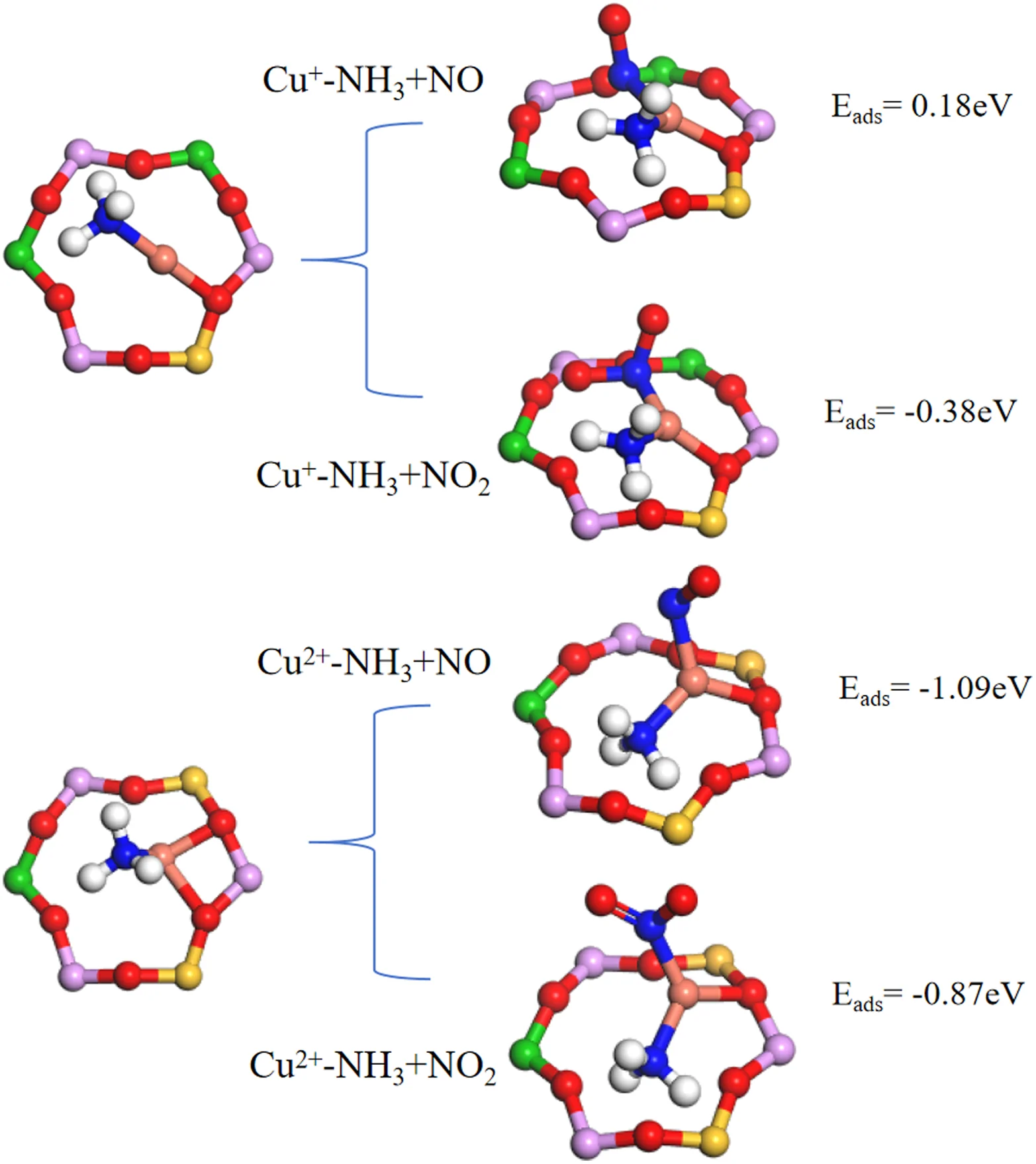
The structure and adsorption energy of NO or NO_2_ co-adsorbed with NH_3_ on Cu^+^ and Cu^2+^.

**Fig. 10:**
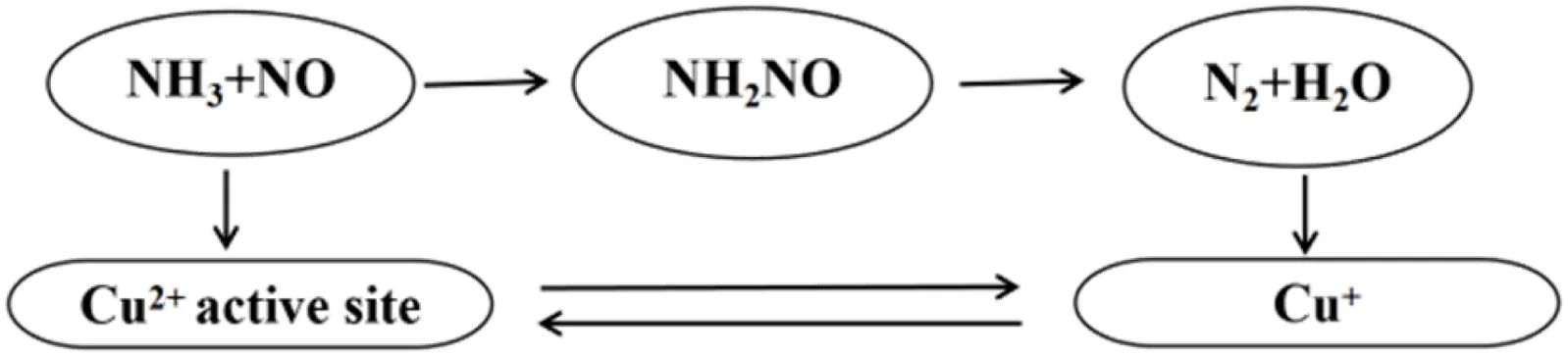
The reaction mechanism of NH_3_-SCR on Cu_*x*_-SAPO-34 catalysts.

## Conclusion

Cu_*x*_-SAPO-34 catalysts with various Cu loadings were synthesized *via* a two-step ion-exchange method, and the SCR reaction mechanism was elucidated from the viewpoints of microstructure, active sites, and reaction pathways. The main conclusions are as follows:

(1) The Cu_4_-SAPO-34 catalyst exhibits good low-temperature activity. The conversion rate of NO_*x*_ reaches over 70% at 130 °C and increases with the rise in temperature, reaching a peak of 98.66% at 170 °C. It begins to decline gradually from 360 °C.

(2) NH_4_-SAPO-34 has a large specific surface area, which is beneficial for the uniform loading of the active component Cu ions on the molecular sieve. With an increase in copper content, the specific surface area exhibits a volcanic trend (first increasing, then decreasing), as the excessive copper species aggregate on the support and block the pore channels of the molecular sieve.

(3) The XRD diffraction peaks of all catalyst samples maintained the original chabazite (CHA) structure of the H-SAPO-34 molecular sieve, indicating that the Cu loading and calcination process did not damage the framework structure of the molecular sieve.

(4) As the Cu doping ratio increases, the signal intensity of the Cu 2p characteristic peak gradually increases, indicating a progressive rise in the relative surface concentration of Cu^2+^ on the catalyst.

(5) Cu_4_-SAPO-34 has the highest total amount and strength of acidic sites, while Cu_6_-SAPO-34 has the weakest, indicating that an excessive Cu loading in this system can cover or neutralize some acidic sites, leading to a decrease in acidity.

(6) The results of the adsorption energy calculations indicate that the adsorption of NH_3_ is the first step of the entire reaction, and it was also found that the Cu^2+^ site is the active site of the reaction.

## Author contributions

Conceptualization: He Huang, Zifei Ni. Methodology: He Huang. investigation: He Huang. Simulation analysis guidance: Heyuan Tian. Data curation: Zifei Ni. Writing – original draft: He Huang. Writing – review and editing: Shujun Yan. Project administration: Shujun Yan.

## Conflicts of interest

There are no conflicts to declare.

## Data Availability

All supporting information is contained within the manuscript.
